# Molecular docking analysis of the tumor protein beta arrestin-1 with oxadiazole compounds

**DOI:** 10.6026/97320630019111

**Published:** 2023-01-31

**Authors:** Vipra Sharma, Gayathri Rengasamy, Surya Sekaran, Kavitha Sankaran, Vishnu Priya Veeraraghavan, Rajalakshmanan Eswaramoorthy

**Affiliations:** 1 Department of Biochemistry, Saveetha Dental College and Hospitals, Saveetha Institute of Medical and Technical Sciences (SIMATS), Saveetha University, Chennai 600077, India; 2 Department of Biomaterials (Green lab), Saveetha Dental College and Hospital, Saveetha Institute of Medical and Technical Sciences (SIMATS), Saveetha University, Chennai 600077, India

**Keywords:** Oral cancer, beta arrestin-1 protein, oxadiazole derivatives, molecular docking, ADMET

## Abstract

Beta arrestins are a family of adaptor proteins that help in the regulation of signaling and trafficking of various G protein coupled receptors (GPCRs). Six oxadiazole derivatives taken from literature are analyzed for anti-cancer properties. The toxicity profiles of all the drugs were similar to Tamoxifen used as control. Data shows that compounds 2, 4, and 6 exhibited comparably significant molecular interactions with the cancerous protein for further consideration.

## Background:

Beta arrestins are cytoplasmic proteins which showcase maximum expression throughout the human body. They regulate the G protein coupled receptors and come out as important nodes in cellular signaling pathways [1]. Beta arrestin-1 is part of the arrestin family of proteins, which is recruited in the nucleus specifically regulating the transactivation of the epidermal growth factor receptors (EGF). MAPK pathway plays an important role in the physiological processes including the tumorigenesis and further development of tumor / cancer. The beta arrestin-1 protein, which is scaffold protein, is associated with the MAPK cascade and downstream targeting of the various GPCRs, therefore, promoting the progression of cancer. Previous reviews suggested that beta arrestin1 interaction with several other signaling pathways such as Wnt, NF-kB, mitogen‑activated protein kinase/extracellular signal regulated kinase, etc. signaling pathways leads to cellular migration, invasion, transmission of apoptotic survival signals affecting several characteristics of the tumor such as the drug resistance, metastatic potential and tumor growth rate [2]. Oral cancer has become a global cancer for a decade now. Nuclear receptors act as the transcription factors regulating various biological processes such as growth, differentiation and metabolism [3]. Deregulation of the various NRs lead to alteration leading to metabolism epigenetics changes, impaired signaling by proteins like beta arrestin-1 [4]. The receptors present in the endothelium cells are targeted using various agonists and lead to inhibition of the nuclear transcription [5]. In the present study, 1, 3, 4 - Oxadiazole compounds/ derivative / isomers are utilized as anti-cancer drugs (Ligands) against beta arrestin-1 protein. These derivatives are taken under consideration due to anti proliferative activity affecting various mechanisms such as inhibition of growth factors, enzymes, kinases and others [6]. These drugs inhibit the production of Tyrosine kinase which if overexpressed or overproduced could cause metastasis and angiogenesis of neoplasm; this inhibition causes cancer regression [7]. Further, in silico analysis of the Beta arrestin-1 is done to find out the 9 best conformations which showcase excellent ligand- protein interaction and act as an antagonist. The pharmacological parameters such as drug likeness, ADME properties and toxicity are analyzed providing insights towards the ligands taken under consideration [8].

## Materials and Method:

## Preparation of ligands:

**Figure 1:**
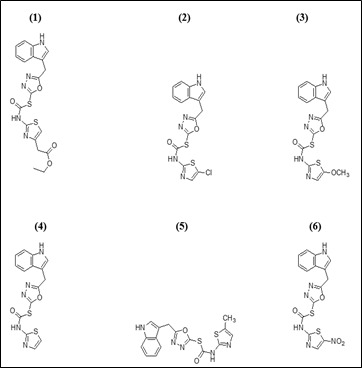
2D Structures of the oxadiazole compounds (1-6).

2D structures (mol.) of oxadiazole compounds (1-6) were drawn using the ChemDraw 16.0 software (Figure 1). During the optimization method, the software Chem3D was employed and all parameters were selected in order to achieve a stable structure with the least amount of energy. The structural optimization approach was used to estimate the global lowest energy of the title chemical. Each molecule's 3D coordinates (PDB) were determined using optimized structure.

##  Preparation of the Beta arrestin-1 protein:

**Figure 2:**
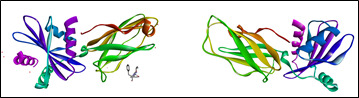
3D structure of tumor protein beta arrestin-1 of *Homo sapiens* and prepared protein.

The 3D crystal structure (Figure 2) of the receptor molecule, beta arrestin1 was downloaded from the protein data bank (PDB ID 2IV8). As per the standard protocol, the preparation of protein was done. Subsequently attached ligands were detached; also, water molecules and co factors were eliminated. Further, the addition of polar hydrogen, and kollman charges were added using the Auto preparation of the target protein file auto dock (MGL tools 1.5.7.).

##  Autodock Vina:

A graphical user interface program called as Auto Dock 4.2.6 was utilized for setting the grid box and subsequently do the docking simulations. Several, different docking pockets and poses were analyzed, but finally the grid was generated with best 9 conformational poses, these structures had the most favorable (least) free binding energy, leading to significant interactions between the ligand and the prepared optimized protein.

##  Drug likeness and toxicity predictions:

These predictions help to understand the drug efficiency and provide insight over the studied ligand properties, analyzing if it is an orally active drug or not. The prediction is done on the basis of Lipinski's rule of five. The chemical structures of the compounds (1-6) were converted to their canonically simplified molecular structures. SwissADME tool was used for the estimation of the pharmacokinetic parameters. The ligands were subjected to the Lipinski's screening using the SwissADME and pre ADMET predictor. Organ toxicities and toxicological endpoints of the ligands and their LD50 values were predicted using ProTox II online server.

##  Statistical analysis:

One way ANOVA was used for statistical analysis. The clinically proven drugs are used as a control and the results are compared. The significance of the results was found to be p< 0.05 

## Results:

## Molecular docking interaction of oxadiazole compounds against tumor protein beta arrestin-1 of *Homo sapiens*:

**Figure 3:**
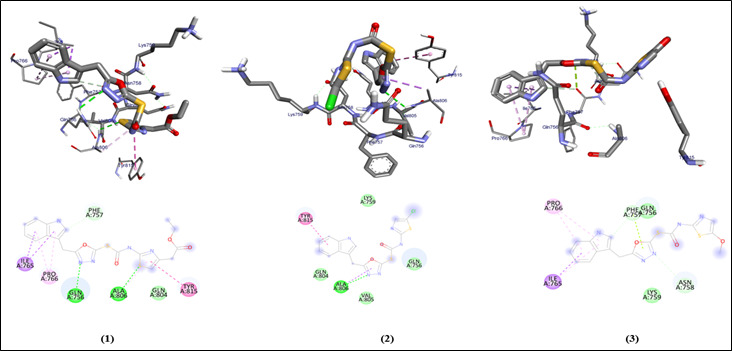
Molecular docking analysis of oxadiazole compounds (1-3) against beta aresstin-1 protein from *Homo sapiens*

**Figure 4:**
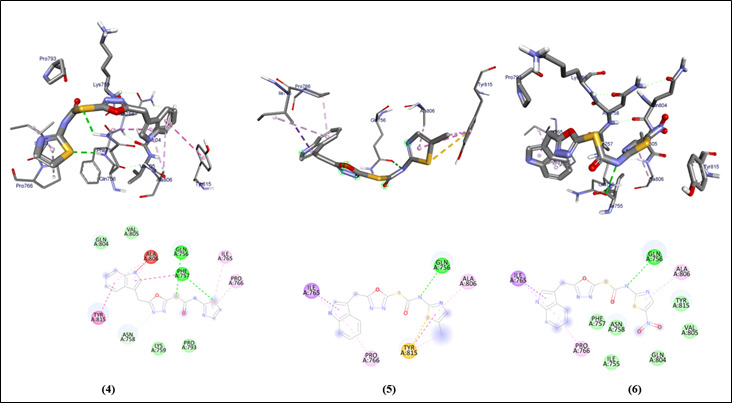
Molecular docking analysis of oxadiazole compounds (4-6) against beta aresstin-1 protein from *Homo sapiens*

The 3D structures of the original and prepared protein show structural conformational differences. The 2D structures of all the ligands are also provided with the similar oxygen , sulfur and nitrogen groups present. The different interactions of the Van der waals , conventional hydrogen bonds , Pi sulfur , alkyl and Pi alkyl bonds are observed (Figure 3, Figure 4) in all the derivatives and the standard drug which are further analyzed using the amino acid residual interaction and providing with the molecular docking scores of the prepared protein (PDB ID:2IV8) and ligands, concluding that the docking affinity of the ligands ( -5.1 , -5.6 , -5 , -6.4 , -5.9 and -6.1 kcal/mol ) was nearest to the docking score of Tamoxifen drug (Table 1). Specific amino acid interactions have been presented by the compounds and the standard drugs. The H bonds were not observed in the standard drugs.

## SwissADME and Lipinski's rule of five:

The oxadiazole derivative ligands, acted as CY2D inhibitors and also obeyed the Lipinski's rule of five, the compounds had low GI absorption and had presented no blood brain barrier permeation (Table 2). The ligands did not act as the Pgp substrates, whereas the standard drugs did act as substrates. The molecular weight of the ligands was less than 500, but the molecular weights of the standard drugs doxorubicin (543.52) and Paclitaxel (853.91) was higher than the standard value. Tamoxifen molecular weight is comparably lower than the other drugs (371.51). The bioavailability score of all the ligands and Tamoxifen (Standard drug) is 0.55 (Table 3).

## Toxicity profiling:

The PROTOX II value signified the toxicity of all the drugs taken under consideration, observation was that only compound 1 had inactive carcinogenicity, other ligands exhibited active carcinogenicity (Table 4). LD50 values analysis of the ligands, the lowest values were of compounds 2, and 5 (300 mg/kg).

## Discussion:

To study the interaction and binding affinity between the beta arrestin-1 protein and oxadiazole compounds in a 3D fashion, compounds were docked within the binding sites of the protein [9]. For the specific ligand protein interaction, the removal of the co crystallized ligands already attached to the existing / original protein was done, for observing the compound's interactions [10]. There were various functional groups, A chain, B chain, heterogenous chains suspending with the original protein [11], the removal of these structures increased the binding affinity of the compound, and the compound synthesized had free active sites and H bonds, Kollman charges were attached to provide stability to the structure [12]. The SwissADME predictions indicating no violations of the Lipinski's rule of five suggested that the ligands synthesized showcased proper absorptions and were orally active substances and on the basis of this observation the Veber rule was also not violated, concluding that a better polar surface area and rotatable bonds were found in the compounds (1-6). [13], [14]. The log Kp values lie between - 6.59 to -3.5 cm/s and molecular docking scores of specific compounds such as 4, 5 and 6 suggested best protein - ligand interactions, also the LD50 values of the compounds 4, 5, and 6 [15,16]. Tamoxifen exhibits best interaction as a standard with beta arrestin-1 as it is having the lowest docking score leading to maximum stability of the ligand with the protein (Beta arrestin). [17,18] The H bonding between the amino acids is of 756, 757 showcasing the H bond stabilization between them. The cariogenic activity of 2, 3, 4, and 6 is of significance to Tamoxifen. All the compounds are potential inhibitions towards CY2D, and the ligands have low GIT absorption, no blood brain permeation, due to the orally active nature of the ligands. Also, the spatial, structural orientation analysis suggested that the 4, 5, and 6 are significantly similar to the standard drug chosen (Tamoxifen) [19, 20]. The potential anti-cancer drugs chosen from the six compounds / ligands were 4, 5, and 6. These drugs could act as potent nuclear transcription inhibition leading to reduction of oral cancer [21, 22]. Also, toxicity property analysis showcases the significant effect of the compounds chosen and concludes that hepatotoxicity is shown by them but no immunotoxicity and mutagenicity.

## Conclusion:

We document the molecular docking analysis of the tumor protein beta arrestin-1 with oxadiazole compounds. Data shows that compounds 2, 4, and 6 exhibited comparably significant molecular interactions with the cancerous protein for further consideration.

## Figures and Tables

**Table 1:** Molecular docking scores and residual amino acid interactions of Oxadiazole compounds (1-6) against Beta Aresstin-1 protein of *Homo sapiens* (PDB ID - 2IV8).

Ligands	Docking scores/Affinity	H-bond	Amino Acid Residual interactions	
			Hydrophobic/Pi-Cation	Van dar Waals
1	-5.1	Gln -756,	Ile - 765, Pro-766, Tyr-815, Phe-757	Gln - 804, Val - 805
		Ala - 806		
2	-5.6	Ala-806	Tyr-815	Gln-804, Val - 805, Gln-756, Lys-759
3	-5		Pro-766, Ile -765, Asn-758, Phe-757, Gln - 756	Lys-759
4	-6.4	Phe-757, Gln-756	Tyr-815, Ala-806, Ile-765, Pro-766	Gln-804, Val-805, Lys-759, Pro-793
5	-5.9	Gln - 756	Ile - 765, Pro-766, Tyr-815, Al-806	
6	-6.1	Gln -756	Ile - 765, Pro - 766, Ala-806	Phe -757, Asn-758, Ile - 755, Gln - 804, Val-805, Tyr-815,
Doxorubicin	55.9		Phe-757, Lys-759, Asn-758, Gln-804, Gln-756, Ile-765, Pro-793	Val-805, Tyr-815, Ala-806
Paclitaxel	141.2		Asn-802, Met-797, Gln-804, Asn-758, Phe-757, Gln-756, Lys-759, Ile-756, Pro-793	Ser-761, Val-805, Ala-806, Ile-755, Pro-766, Gly-763, Val-764, Gly-792, Ser-817
Tamoxifen	7		Ala-806, Gln-756, Pro-766, Lys-759,	Tyr-815, Ile-765, Pro-766, Phe-757, Asn-758,

**Table 2:** SwissADME values of selected oxadiazole compounds (1-6)

Compound	log Kp (cm/s)	GI absorption	BBB permeant	Pgp substrate	CYP1A2 inhibitor	CYP2C19 inhibitor	CYP2C9 inhibitor	CYP2D6 inhibitor	CYP3A4 inhibitor
1	-6.59	Low	No	No	No	Yes	Yes	No	Yes
2	-5.73	Low	No	No	Yes	Yes	Yes	No	Yes
3	-6.16	Low	No	No	Yes	Yes	Yes	No	Yes
4	-6.2	Low	No	No	Yes	Yes	Yes	No	Yes
5	-6	Low	No	No	Yes	Yes	Yes	No	Yes
6	-6.36	Low	No	No	No	Yes	No	No	Yes
Doxorubicin	-8.71	Low	No	Yes	No	No	No	No	No
Paclitaxel	-8.91	Low	No	Yes	No	No	No	No	No
Tamoxifen	-3.5	Low	No	Yes	No	Yes	No	Yes	No

**Table 3:** Lipinski and Veber rules of selected oxadiazole compounds (1-6)

Compound	MW	iLogP	HBD (nOHNH)	HBA (nON)	nrotb	MR	TPSA	Lipinski #violations	Bio availability score
Lipinski*	≤500	≤5	≤5	≤10	≤10	-	-		
Veber**	-	-	-	-	-	-	≤ 140		
1	443.5	2.36	2	7	10	113.09	176.54	0	0.55
2	391.86	2.45	2	5	6	97.43	150.24	0	0.55
3	387.44	2.28	2	6	7	98.91	159.47	0	0.55
4	357.41	2.16	2	5	6	92.42	150.24	0	0.55
5	371.44	2.45	2	5	6	97.38	150.24	0	0.55
6	402.41	1.56	2	7	7	101.24	196.06	0	0.55
Doxorubicin	543.52	2.16	6	12	5	132.66	206.07	3	0.17
Paclitaxel	853.91	4.51	4	14	15	218.96	221.29	2	0.17
Tamoxifen	371.51	4.64	0	2	8	119.72	12.47	1	0.55

**Table 4:** Toxicity profile of selected oxadiazole compounds (1-6)

			Toxicity				
Compound	^a^LD_50_ (mg/kg)	Class	HEPATOTOXICITY	CARCINOGENICITY	IMMUNOTOXICITY	MUTAGENICITY	CYTOTOXICITY
1	1190mg/kg	4	Active	Inactive	Active	Inactive	Inactive
2	300mg/kg	3	Active	Active	Inactive	Inactive	Inactive
3	1000mg/kg	4	Active	Active	Inactive	Inactive	Inactive
4	1000mg/kg	4	Active	Active	Inactive	Inactive	Inactive
5	300mg/kg	3	Active	Active	Inactive	Inactive	Inactive
6	1350mg/kg	4	Active	Active	Inactive	Active	Inactive
Doxorubicin	205mg/kg	3	Inactive	Inactive	Active	Active	Active
Paclitaxel	134mg/kg	3	Inactive	Inactive	Active	Inactive	Active
Tamoxifen	1190mg/kg	4	Active	Inactive	Active	Inactive	Inactive
